# Subcellular localization of the voltage-gated K+ channel EGL-36 , a member of the KV3 subfamily, in the ciliated sensory neurons in *C. elegans*

**DOI:** 10.17912/micropub.biology.000367

**Published:** 2021-03-05

**Authors:** Sebiha Cevik, Oktay I. Kaplan

**Affiliations:** 1 Rare Disease Laboratory, School of Life and Natural Sciences, Abdullah Gul University

## Abstract

Delineated as the first cellular organelle in 1675 by Antonie van Leeuwenhoek, cilia did not receive much attention until the 2000s, when it became apparent that cilia played a key role in the development of embryos, a variety of signaling pathways. Therefore, collective efforts by many scientists have led to the identification of many novel ciliopathy and cilia genes, while we are still far from disclosing the complete components of cilia.**Here we used the ciliated sensory neurons in *C. elegans *as a model system that revealed the voltage-gated K+ channel EGL-36 (a member of the *Shaw *subfamily) as a new component associated with cilia. The confocal microscopy examination of fluorescence tagged EGL-36 together with ciliary (IFT-140) or transition zone (MKS-6) markers reveal that EGL-36 is only expressed in subsets of the ciliated sensory neurons, where it partially overlaps with the basal body signals and predominantly localizes to the periciliary membrane compartment. This expression pattern along with studies of *egl-36* gain-of-function variants indicates that *egl-36* is not essential for ciliogenesis in *C. elegans*. Our data identify the voltage-gated K+ channel EGL-36 as a new cilia-associated protein, and future studies should reveal the functional significance of EGL-36 in cilia biogenesis.

**Figure 1. EGL-36 is localized to the region proximal to the ciliary base in C. elegans:**
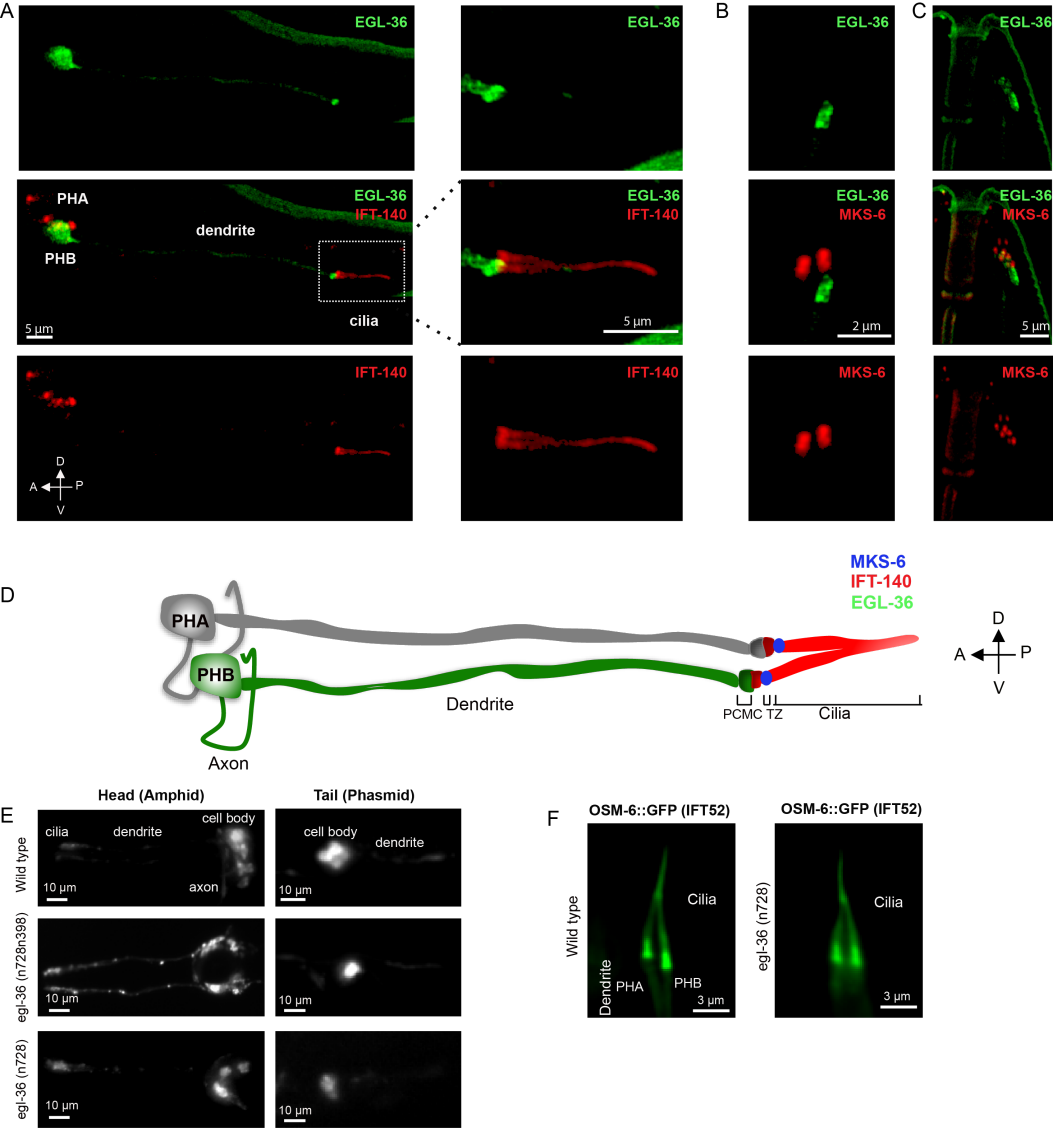
**A)**EGL-36::GFP specifically localizes at the cell soma and a compartment in the distal dendrite, just proximal to the ciliary base, in PHB not in PHA sensory neurons (left and middle columns). Shown are colocalization of EGL-36::GFP and IFT-140::mCherry, a ciliary marker, (a single copy). Scale bar: 5 μm, **B and C)** Co-expressions of EGL-36::GFP and MKS-6::mCherry, a transition zone marker (a single copy), is displayed in head (C, right column) and tail sensory neurons (B middle columns) in *C. elegans*. EGL-36::GFP is visible in PHB, not in PHA,and does not enter into cilia **D)** An illustration showing the localization of EGL-36 in relation to cilia in the tail sensory neurons (PHA/PHB). IFT-140 and MKS-6 stain the entire ciliary axoneme and the transition zone, respectively. PCMC and TZ denote the periciliary membrane compartment and transition zone, respectively. A, anterior; P, posterior; D, dorsal; V, ventral. (L) Ventral view. **E)** Fluorescence images display lipophilic fluorescent dye uptake for wild type, *egl-36 (n728)*, and *egl-36 (n728n398)*. No obvious Dye uptake defects for these mutants **F)** Localizations of OSM-6::GFP (IFT52) in PHA/PHB cilia were shown in wild type and *egl-36 (n728)* (no obvious phenotype).

## Description

*C. elegans* EGL-36 displays extensive similarity to human KCNC proteins, the conserved potassium channel subfamily KV3 (the Shaw subfamily). This potassium channel subfamily contains four genes KCNC1, KCNC2, KCNC3, and KCNC4 encoding KV3.1 (Shaker), KV3.2 (Shab), KV3.3 (Shaw), and KV3.4 (Shal), respectively. Previous studies have demonstrated that *C. elegans* EGL-36 is expressed in the egg-laying muscles, ventral cord neurons, and the subset of ciliated sensory neurons including ADEL, ADER, PHB through its subcellular localization in the ciliated sensory neurons remains unknown (Elkes *et al.* 1997; Johnstone *et al.* 1997). To observe the subcellular localization of EGL-36 in the ciliated sensory neurons in *C. elegans*, we tagged green fluorescent protein (GFP) at the C-termini of EGL-36 with a cilia-specific promoter. We observed overexpressed EGL-36::GFP in the subsets of sensory neurons in the head and tail including PHB sensory neurons in the tail but not in the PHA sensory neuron, which is in line with previous findings (Elkes *et al.* 1997; Johnstone *et al.* 1997) (**Figure 1A**). Confocal laser scanning microscopy shows that EGL-36::GFP is predominantly located at the base of the cilium.The EGL-36::GFP signal can be found in the neuronal cell body, with weak dendrite staining, where they may function in the dendritic excitability (**Figure 1A**).

Cilia have several sub-compartments including the basal body, the transition zone, the middle segment, and the ciliary tip. We further examined the exact localization of GFP-tagged EGL-36 relative to these compartments. To this end, we used the transition zone marker MKS-6/CC2D2A and the cilia marker IFT-140 (intraflagellar transport 140), and co-expressed EGL-36::GFP with either of these markers (Qin *et al.*. 2001; Williams *et al.*. 2011; Prevo *et al.* 2015 Mijalkovic *et al.* 2018). Our microscopy analysis revealed that some of EGL-36::GFP signal was, however, found to overlay with the proximal zone of IFT signal (marked with IFT-140::mCherry), while the majority of EGL-36:: GFP signal was predominantly localized in a compartment, proximal to the ciliary base (**Figure 1A**). This strong EGL-36 staining likely corresponds to the periciliary membrane compartment (PCMC) (Kaplan *et al.* 2012). There is no overlay of EGL-36::GFP signal with MKS-6::mCherry (**Figure 1B and C**).

We next went on characterizing potential ciliary functions of EGL-36 in *C. elegans*, we therefore obtained two mutants for *egl-36* including *egl-36(n728)* and *egl-36(n728n398). egl-36(n728)* mutantcontains a missense variant, conversion of glutamic acid at the 142^nd^ position of EGL-36 to lysine (p.E142K). EGL-36(p.E142K) represents a gain of function variant of *egl-36*, which displays defects in egg-laying in *C. elegans.* On the other hand, proline to serine conversion (p.P439S) in *egl-36* suppresses the egg-laying defect of p.E142K variant (Elkes et. al., 1997; Johnstone et. al., 1997). *egl-36(n728n398)]* mutant contains both p.E142K and p.P439S variants. First, we performed the DiI-filling assay, which has been used to test ciliary structural defects in *C. elegans*. The lipophilic fluorescent dye is not taken up by *C. elegans* with abnormal cilia structures, while wild types always stain their ciliated sensory neurons with this fluorescent dye (Herman and Hedgecock 1990). The dye analysis coupled with microscopy imaging revealed that similar to wild type, both *egl-36* mutants fill up their ciliated sensory neurons with the lipophilic fluorescent dye, suggesting that *egl-36* mutants have no gross cilia abnormalities in *C. elegans* (**Figure 1E**). We next examined the cilia morphologies of phasmid (tail) sensory neurons in *egl-36(n728*) mutant. We generated transgenic strains expressing OSM-6/IFT52::GFP in *egl-36(n728)* mutants, and our microscopy analysis revealed that PHB (tail) displayed no abnormality in cilia morphology in *egl-36(n728)* mutants (**Figure 1F**).

## Methods

***C. elegans* strains and maintenance**

All strains used were grown in the nematode growth medium (NGM) at 20°C as previously described (Brenner, 1974).

**Generation of transgenic strains with microinjection**

The transgenic strain *OIK904, N2;turEx12[Parl-13::EGL-36::GFP::unc-54 3`UTR+pRF4]* was generated with microinjection of the *Parl-13::EGL-36 (cDNA)::GFP::unc-54 3` UTR* construct (25 ng/μl) along with the *rol-6* co-injection marker (50 ng/μl) by our laboratory.

**Dye-filling assays**

Dye filling assay previously described was used (Cevik *et al.*. 2010; Herman and Hedgecock 1990).

**Fluorescent and Confocal microscopy**

Fluorescent images for the dye assay were obtained by a fully automated upright microscope system (Leica DM6 B ) with a Plan ApoChromat 100x/1.40 NA and an electron-multiplying charge-coupled device camera (Andor iXon Ultra 897 EMCCD camera and iQ3.6.2 Andor software) that is attached to the microscope while confocal images were acquired with a laser-scanning confocal inverted microscope (Zeiss LSM 900 with Airyscan 2 and ZEN 3 Blue edition software) with a Plan ApoChromat 63x/1.40 NA objective.

## Reagents

Strains and plasmid are available upon request to sebiha.cevik(at)agu.edu.tr

**Table:** 

**Strain**	**Genotype**	**Available from**
N2	*Caenorhabditis elegans*	CGC
MT1540	*egl-36(n728) X*.	CGC
KP100	*egl-36(n728n398) X.*	CGC
SP2101	*osm-6(p811);mnIs17[osm-6::gfp; unc-36(+)*	CGC
OIK912	*egl-36 (n728)X.; mnIs17[osm-6::gfp; unc-36(+)*.	This study
OIK904	*turEx12[Parl-13::EGL-36::GFP::unc-54 3`UTR+pRF4]*	This study
EJP81	*vuaSi24 [pBP43; Pche-11::che-11::mCherry; cb-unc-119(+)]II; unc-119(ed3) III; che-11(tm3433)V. (*This is referred IFT-140 throughout to the text)	Peterman Lab
DAM954	*vuaSi21[pBP39; Pmks-6::mks-6::mCherry; cb-unc-119(+)]II.;mks-6(gk674) I (*This is referred MKS-6 throughout to the text)	Dammermann Lab
OIK952	*N2;turEx12[Parl-13::EGL-36::GFP::unc-54 3`UTR+pRF4];vuaSi21[pBP39; Pmks-6::mks-6::mCherry; cb-unc-119(+)]II.*	This study
OIK953	*N2;turEx12[Parl-13::EGL-36::GFP::unc-54 3`UTR+pRF4];vuaSi24 [pBP43; Pche-11::che-11::mCherry; cb-unc-119(+)]II; unc-119(ed3) III; che-11(tm3433)V.*	This study
**Plasmids**	**Genotype**	**Description**
OK133	P*arl-13*::*EGL-36 (cDNA) ::GFP:*:*unc-54* 3`UTR	Inserted *Parl-13* (300 bp)- *egl-36* (cDNA) with SphI and Age I restriction enzymes by Sunybiotech
pRF4	*rol-6*(*su1006*)	(Mello C *et al.* 1991)

## References

[R1] Brenner, S. 1974. The genetics of <i>Caenorhabditis elegans</i>. Genetics. 77: 71–94. 10.1093/genetics/77.1.71 PMC1213120 4366476

[R2] Cevik, S., Hori, Y., Kaplan, O.I., Kida, K., Toivenon, T., Foley-Fisher, C., Cottell, D., Katada, T., Kontani, K., Blacque, O.E. 2010. Joubert syndrome Arl13b functions at ciliary membranes and stabilizes protein transport in <i>Caenorhabditis elegans</i>. Journal of Cell Biology. 188: 953–969. 10.1083/jcb.200908133 PMC2845074 20231383

[R3] Elkes, D.A., Cardozo, D.L., Madison, J., Kaplan, J.M. 1997. EGL-36 <i>Shaw</i> Channels Regulate <i>C. elegans </i>Egg-Laying Muscle Activity. Neuron. 19: 165–174. 10.1016/s0896-6273(00)80356-6 9247272

[R4] Herman, R.K., Hedgecock, E.M. 1990. Limitation of the size of the vulval primordium of <i>Caenorhabditis elegans</i> by <i>lin-15</i> expression in surrounding hypodermis. Nature. 348: 169–171. 10.1038/348169a0 2234080

[R5] Johnstone, D.B., Wei, A., Butler, A., Salkoff, L., Thomas, J.H. 1997. Behavioral Defects in <i>C. elegans egl-36 </i>Mutants Result from Potassium Channels Shifted in Voltage-Dependence of Activation. Neuron. 19: 151–164. 10.1016/s0896-6273(00)80355-4 9247271

[R6] Kaplan OI, Doroquez DB, Cevik S, Bowie RV, Clarke L, Sanders AA, Kida K, Rappoport JZ, Sengupta P, Blacque OE (2012). Endocytosis genes facilitate protein and membrane transport in C. elegans sensory cilia.. Curr Biol.

[R7] Mello CC, Kramer JM, Stinchcomb D, Ambros V (1991). Efficient gene transfer in C.elegans: extrachromosomal maintenance and integration of transforming sequences.. EMBO J.

[R8] Mijalkovic, J., van Krugten, J., Oswald, F., Acar, S., Peterman, E.J.G. 2018. Single-Molecule Turnarounds of Intraflagellar Transport at the<i> C. elegans</i> Ciliary Tip. Cell Reports. 25: 1701-1707. 10.1016/j.celrep.2018.10.050 30428341

[R9] Prevo, B., Mangeol, P., Oswald, F., Scholey, J.M., Peterman, E.J.G. 2015. Functional differentiation of cooperating kinesin-2 motors orchestrates cargo import and transport in <i>C. elegans</i> cilia. Nat Cell Biol.17: 1536–1545. 10.1038/ncb3263 26523365

[R10] Qin, H., Rosenbaum, J.L., Barr, M.M. 2001. An autosomal recessive polycystic kidney disease gene homolog is involved in intraflagellar transport in <i>C. elegans </i>ciliated sensory neurons. Current Biology. 11: 457–461. 10.1016/s0960-9822(01)00122-1 11301258

[R11] Williams, C.L., Li, C., Kida, K., Inglis, P.N., Mohan, S., Semenec, L., Bialas, N.J., Stupay, R.M., Chen, N., Blacque, O.E., Yoder, B.K., Leroux, M.R. 2011. MKS and NPHP modules cooperate to establish basal body/transition zone membrane associations and ciliary gate function during ciliogenesis. Journal of Cell Biology. 192: 1023–1041. 10.1083/jcb.201012116 PMC3063147 21422230

